# Emotion Effects on Timing: Attention versus Pacemaker Accounts

**DOI:** 10.1371/journal.pone.0021829

**Published:** 2011-07-20

**Authors:** Ming Ann Lui, Trevor B. Penney, Annett Schirmer

**Affiliations:** 1 Department of Psychology, National University of Singapore, Singapore, Singapore; 2 Institute of Cognitive Neuroscience, National Central University, Jhongli City, Taiwan; Duke University, United States of America

## Abstract

Emotions change our perception of time. In the past, this has been attributed primarily to emotions speeding up an “internal clock” thereby increasing subjective time estimates. Here we probed this account using an S1/S2 temporal discrimination paradigm. Participants were presented with a stimulus (S1) followed by a brief delay and then a second stimulus (S2) and indicated whether S2 was shorter or longer in duration than S1. We manipulated participants' emotions by presenting a task-irrelevant picture following S1 and preceding S2. Participants were more likely to judge S2 as shorter than S1 when the intervening picture was emotional as compared to neutral. This effect held independent of S1 and S2 modality (Visual: Exps. 1, 2, & 3; Auditory: Exp. 4) and intervening picture valence (Negative: Exps. 1, 2 & 4; Positive: Exp. 3). Moreover, it was replicated in a temporal reproduction paradigm (Exp. 5) where a timing stimulus was preceded by an emotional or neutral picture and participants were asked to reproduce the duration of the timing stimulus. Taken together, these findings indicate that emotional experiences may decrease temporal estimates and thus raise questions about the suitability of internal clock speed explanations of emotion effects on timing. Moreover, they highlight attentional mechanisms as a viable alternative.

## Introduction

In an effort to distinguish man from beast, ancient philosophers dissociated thought from passion and attributed thought to the workings of the brain and passion to the workings of other bodily organs [1]. Although research since then has demonstrated thought in non-human animals and identified the brain as the substrate for both thought and the subjective feeling state called emotion [2], it is only recently that putative interactions between emotions and various cognitive functions have been subject to significant research (for a review see [3]). This work demonstrated that emotions facilitate perceptual processing by lowering the threshold for awareness [4]; items of emotional relevance to the observer are more likely to be noticed than are neutral items. Emotions have also been found to influence memory [5]; images and verbal information are better remembered if they are emotional as compared to neutral [6]–[8]. Furthermore, there is evidence for emotions affecting language. Positive emotional states enhance the spread of activation among associatively linked words in semantic memory making it easier for individuals to relate these words and to generate ideas based on their perceived relationship [9].

Amidst the growth of studies on the interaction between emotion and cognition, insights into the role of emotions on interval timing have remained limited. Interval timing typically refers to our capacity to monitor and store durations in the hundreds of milliseconds to minutes range and to adjust our behavior based on these durations. For example, we may have a stored temporal representation of how long it takes for someone to answer a knock at their door. Thus, if a person fails to open the door within that time period, we will not continue knocking indefinitely, but will instead move on once the evoked temporal representation has elapsed. Sensitivity to time is a fundamental aspect of many higher order cognitive processes including decision making, memory and language (for a review see [10]). As such it has been a vivid area of research resulting in a number of interval timing models. The most influential model to date is Scalar Expectancy Theory (SET) [11]. The information-processing version of SET [12] postulates clock, memory, and decision stages. When timing the duration of an event, pulses from a pacemaker accumulate to form a temporal representation of the currently elapsing duration. This temporal representation is compared with duration representations previously stored in memory and behavior results when the current interval exceeds some threshold of similarity to the remembered interval. According to this model, interval timing taps a range of cognitive functions including perception, attention, and memory. Thus, when considering the potential influence of emotions on interval timing, the relationship between emotions and these functions may prove critical.

An indication of a role for emotions in time perception comes from both linguistics and timing experiments. The saying “time drags” is typically used in the context of negative or boring events, whereas the saying “time flies” is used in the context of positive or exciting events [13]–[14]. Thus, one may infer that positive emotions lead to an underestimation of duration, whereas negative emotions have the opposite effect. Contrary to this idea, however, experimental research indicates that positive and negative emotions have a similar impact on interval timing. Both apparently lead individuals to overestimate the passing of time. When participants were asked to judge or reproduce the duration of a stimulus, the reported or produced durations were longer when the stimulus was positive or negative as compared to neutral [15]–[19]. Explanations of the effect of emotion on interval timing in these studies rely on pacemaker-accumulator timing models and their presumed clock components. Moreover, they draw on the idea that pacemaker rate is influenced by bodily arousal as reflected by peripheral markers such as heart rate or skin conductance [20]. Accordingly, positive and negative stimuli are believed to increase arousal thus speeding up the pacemaker and causing a greater number of pulses to be accumulated. Therefore, positive and negative stimuli are experienced as longer than equivalent duration neutral stimuli.

While pacemaker rate [21] is a possible mechanism through which emotions can influence timing, it is not the only one. Some researchers propose that emotion effects are mediated by attention [22]–[24]. This proposal rests on evidence that temporal estimates vary as a function of attention [25]–[27]. For example, temporal estimates are shorter if attention is distracted by a concurrent verbal task [28]. The information-processing version of SET accommodates these and similar findings [29]–[30]. It holds attention responsible for whether the mode switch allows transfer of pulses from the pacemaker to the accumulator [29]. This switch may “flicker” or oscillate between the open and closed states during the timing signal [31]–[33]. Hence, when more attention is allocated to the timing signal, a larger number of pulses are accumulated and the duration seems longer as compared to a situation in which less attention is allocated to the timing signal. Thus, if emotional events enhance attention to the timing signal above baseline levels, they may inadvertently contribute to a larger number of pulses being collected and lead to event duration being perceived as longer than the duration of an equally long, but emotionally neutral, event.

Evidence for the later proposition comes from research that highlights the capacity of emotional stimuli to capture and hold attention. For example, visual search studies indicate that participants are faster at detecting an emotional target among neutral distracters and slower at detecting a neutral target among emotional distracters relative to an all neutral baseline [34]. Likewise, experiments using the Posner spatial cueing paradigm [35] revealed that participants are more likely to benefit from valid cues if they are emotional as compared to neutral [36]. Additionally, they are less likely to disengage from invalid emotional as compared to neutral cues [37]. Thus, one may speculate that the attentional advantage gained by encoding emotional as compared to neutral stimuli modulates the accumulation of pacemaker pulses and perceived stimulus duration. If true, timing an emotional event should produce a longer duration estimate because more pulses accumulate than when timing a neutral event. Moreover, the opposite effect should emerge when individuals time a neutral event in the context of an emotional or neutral distracter. The emotional distracter should be more successful in diverting attention away from the target event resulting in shorter duration estimates than in the context of a neutral distracter.

While there is ample evidence of participants overestimating the duration of emotional relative to neutral events [15]–[19], the role of emotional and neutral distracters for the timing of *neutral* events has not yet been explored. To fill this gap, we employed an S1/S2 temporal discrimination paradigm. In this paradigm, a stimulus (S1) was presented and the participant was asked to encode its duration. After a short retention interval, a second stimulus (S2) appeared and the participant indicated whether it was shorter or longer than the first one. During the retention interval, a picture with emotional or neutral content was presented to manipulate the participant's emotion independently of the timing task. If, as proposed previously, emotions affect time perception by modulating pacemaker rate, this paradigm should reveal an overestimation of S2 duration on trials with emotional as compared to neutral task-irrelevant images. Heightened arousal on these trials should cause a higher pacemaker rate and more pulses to be accumulated [38]. If, however, emotions affect time perception by modulating attention, we should observe an underestimation of S2 duration on emotional as compared to neutral trials. A diversion of attentional resources from the monitoring of time to the processing of emotionally relevant information should cause participants to miss pacemaker pulses and thus to perceive the interval as shorter.

## Experiment 1

### Method

#### Participants

Fourteen female college students participated in the present study in fulfillment of an introductory psychology course requirement. Their mean age was 19.6 years (SD = 0.65). They were all right-handed [39] and reported normal or corrected to normal vision.

#### Materials

A filled circle 1.7 cm in diameter was used as the timing stimulus (i.e., S1 and S2). Sixty-four task-irrelevant images were selected from the International Affective Picture System (IAPS [40]). Half the images were negative with a mean arousal rating of 6.15 (range 5.25–7.35) and a mean valence rating of 2.22 (range 1.51–2.95). The remaining pictures were neutral with a mean arousal rating of 2.70 (range 1.76–3.29) and a mean valence rating of 5.10 (range 4.45–6.28). Examples of negative pictures include mutilated body parts, assault, and toilet scenes. Examples of neutral pictures include an elderly man, plants, and building scenes. All stimuli were presented on a black background in the center of a computer screen.

#### Procedure

**Figure 1:**
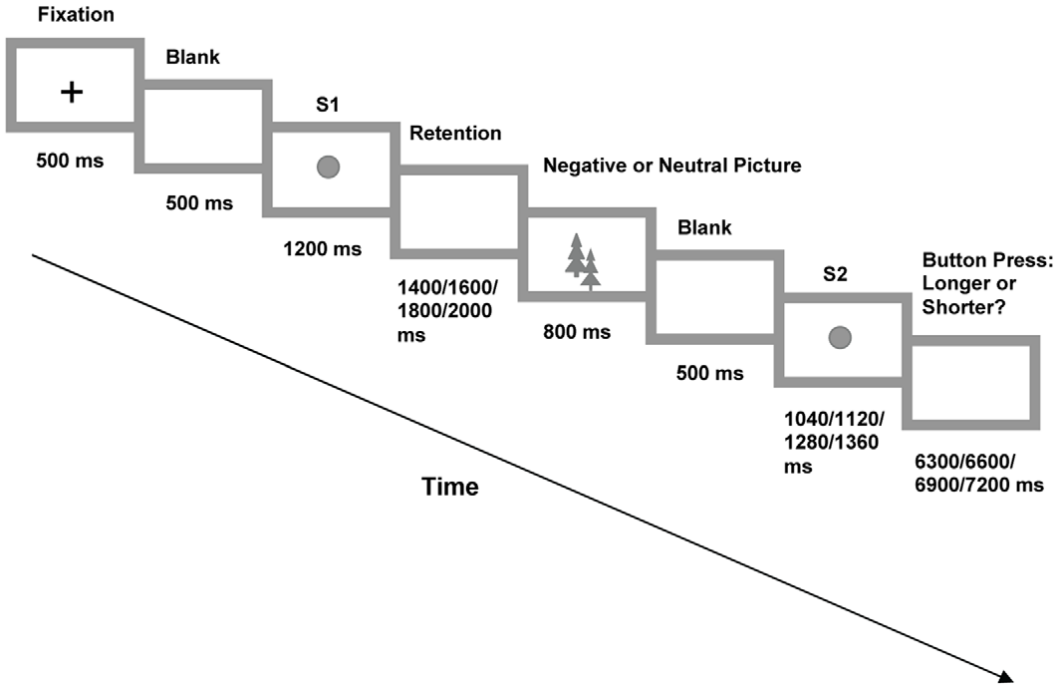
The sequence and duration of stimuli during a trial in Experiment 1 are shown.

Participants were seated in front of a computer screen, instructed on their task and subsequently commenced the experiment. Each trial in the experiment began with the presentation of a fixation cross at screen center for 500 ms followed by a 500 ms blank screen. Then, a circle (S1) appeared for 1200 ms followed by another blank screen of 1400, 1600, 1800, or 2000 ms duration. Next, the participant saw a negative or a neutral picture for 800 ms, followed by a 500 ms blank screen and another circle (S2). S2 was either shorter (i.e., 1040 or 1120 ms) or longer (i.e., 1280 or 1360 ms) than S1. Participants were informed that distracter pictures interleaved every S1/S2 pair and were asked to simply keep looking at the screen. The task instruction was to compare the duration of the two circles (S1 and S2) and to indicate whether S2 was shorter or longer than S1 by pressing one of two buttons on a response box following S2 offset. A new trial started 6300, 6600, 6900, or 7200 ms after the offset of S2 (Fig. 1).

There were 64 trials in the experiment. Each S2 duration was presented 8 times in the negative condition and 8 times in the neutral emotion condition. Trial order was randomized and the assignment of response buttons to response alternatives was counterbalanced across participants.

### Results

**Figure 2:**
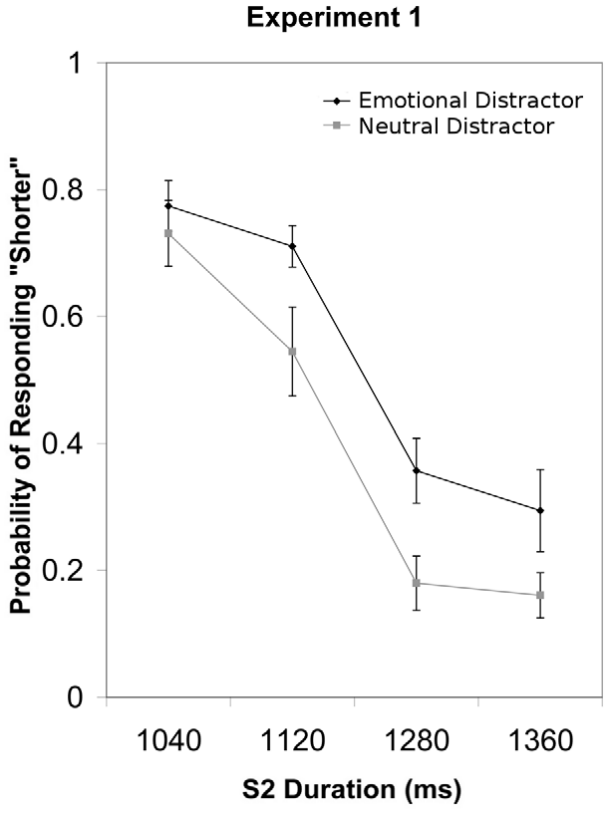
The group mean probability of responding “shorter” and standard error of the mean are plotted for each S2 duration in Experiment 1.

Probability of responding “shorter” was subjected to an ANOVA with Emotion (negative, neutral) and S2 Duration (4 levels) as repeated measures factors. There were significant main effects of S2 Duration (*F*(3, 39) = 73.24, *p*<.001, η^2^_partial_ = .849) and Emotion (*F*(1, 13) = 9.67, *p*<.01, η^2^_partial_ = .426). Participants were more likely to respond “shorter” with decreasing S2 duration and when S2 was preceded by a negative as compared to a neutral picture (Fig. 2). The absence of an interaction between S2 Duration and Emotion (*p*>.1) indicated that the two main effects were independent.

### Discussion

In contrast to previous studies, we found that participants underestimated stimulus duration during emotional as compared to neutral trials. Specifically, participants in the present experiment were more likely to indicate that S2 duration was shorter than that of S1 when S2 was preceded by a negative as compared to neutral picture. Given the nature of the present paradigm, one may speculate that attention, rather than pacemaker rate, accounts for the observed effects. Emotional modulation of attention may have caused participants to accumulate fewer pacemaker pulses such that perceived S2 duration was reduced. However, before such a conclusion can be drawn, alternative accounts require consideration. Specifically, it is possible that the relatively short interval between the picture and S2 accounts for the observed effects. Perhaps the negative picture engaged perceptual mechanisms that led to a transient distraction causing the participant to miss S2 onset.

Evidence for this possibility comes from research employing the “attentional blink” paradigm. In this paradigm, participants are presented with a stream of distracters and one or two targets. Participants are likely to miss a second target if it closely follows the first target [41]–[42]. Based on this evidence researchers argued that attention capturing stimuli elicit an attentional blink, which may last up to 500 ms and which compromises the perception of subsequent information. Although the present study used picture durations of 800 ms, which exceeds the typical stimulus duration of an attentional blink paradigm (<100 ms), the interval between picture offset and S2 onset was only 500 ms. Thus, one may ask whether emotional pictures, due to their capacity for attention capture [43], elicited an extended attentional blink that accounts for the observed effects. Specifically, emotional, but not neutral pictures may have induced an attentional blink causing participants to miss the onset of S2 and thus temporal pulses. To test this possibility, we conducted a second experiment in which the interval between picture offset and S2 onset was 2000 ms. All other parameters were as in Experiment 1.

## Experiment 2

### Method

#### Participants

Fifteen female college students participated in the present study in fulfillment of an introductory psychology course requirement. Their mean age was 18.9 years (SD = 0.64). They were all right-handed [39] and reported normal or corrected to normal vision.

#### Materials and Procedure

In Experiment 2, a blank computer screen was presented for 2000 ms between the offset of the picture and the onset of S2. Nevertheless, the average trial duration was comparable to Experiment 1 as the intervals between trials were shortened to 5300 and 6200 ms. All other parameters were left unchanged.

### Results

**Figure 3:**
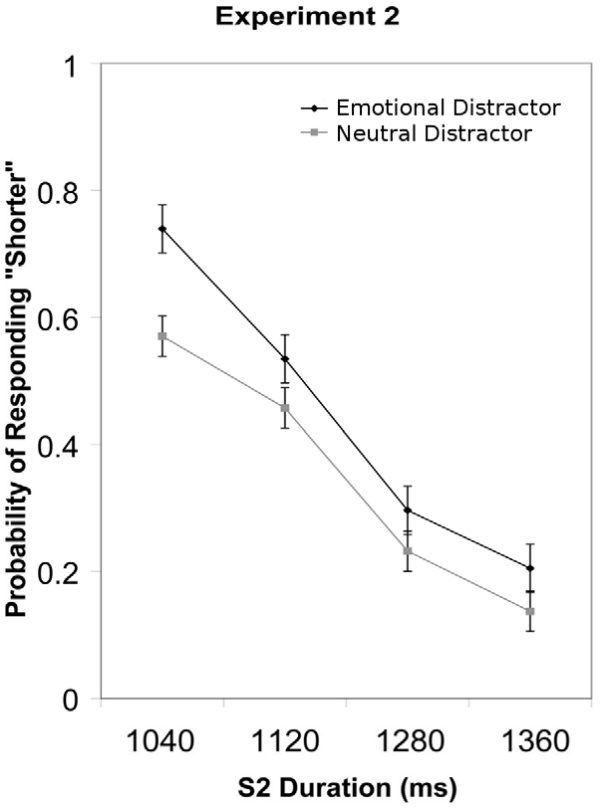
The group mean probability of responding “shorter” and standard error of the mean are plotted for each S2 duration in Experiment 2.

Analysis of the probability of responding “shorter” revealed significant main effects of S2 Duration (*F*(3, 42) = 59.07, *p*<.001, η^2^_partial_ = .808) and Emotion (*F*(1, 14) = 5.15, *p*<.05, η^2^_partial_ = .269). Participants were more likely to respond “shorter” with decreasing S2 duration and when S2 was preceded by a negative as compared to a neutral picture (Fig. 3). S2 Duration and Emotion failed to interact (*p*>.1).

### Discussion

The results of Experiment 2 are similar to those of Experiment 1. Thus, we can exclude the possibility that the effects elicited in Experiment 1 reflect an attentional blink following negative pictures. Instead, we argue that the negative pictures triggered emotional processes that distracted individuals from monitoring the S2 duration, leading to a loss of pacemaker pulses and a temporal underestimation relative to the neutral condition. In a third experiment, we sought to replicate this observation and to determine whether it is specific to negative emotions. Past research revealed consistent evidence for attention capture and distraction by negative emotional events [44]–[45]. However, evidence for a similar role of positive emotions remains rare. Moreover, compared to positive stimuli, potentially threatening stimuli are more readily detected in visual search paradigms and more easily conditioned [34], [43], [46]. Thus, some researchers argue that negative events are more imperative for survival and thus more successful in modulating attention [34], [43], [46]. Given that the emotion effect on timing observed here is postulated to arise from attentional modulation, it may be specific to negative emotions. We tested this idea by conducting a third experiment using positive pictures to elicit an emotional state.

## Experiment 3

### Method

#### Participants

Thirteen female college students participated in the present study in fulfillment of an introductory psychology course requirement. Their mean age was 20.2 years (SD = 1.1). They were all right-handed [39] and reported normal or corrected to normal vision.

#### Materials and Procedure

Experiment 3 was equivalent to Experiment 1 with the exception that the negative IAPS pictures were replaced by positive IAPS pictures. Examples of positive pictures are erotic couple, fireworks, and roller coaster scenes. Positive pictures had a mean arousal rating of 5.72 (range 5.08–6.99) and a mean valence rating of 7.29 (range 6.56–8.22). The mean arousal rating of the positive pictures used in Experiment 3 was significantly lower (*t(1,62)* = 3.25, *p* = .002) than that of the negative pictures used in Experiment 1. Additionally, the absolute differences of the positive and negative pictures' valence ratings from the mean valence rating of the neutral items were compared. The negative pictures were more different from the neutral pictures than were the positive pictures (*t*(1,62) = 7.14, *p*<.001).

### Results

**Figure 4:**
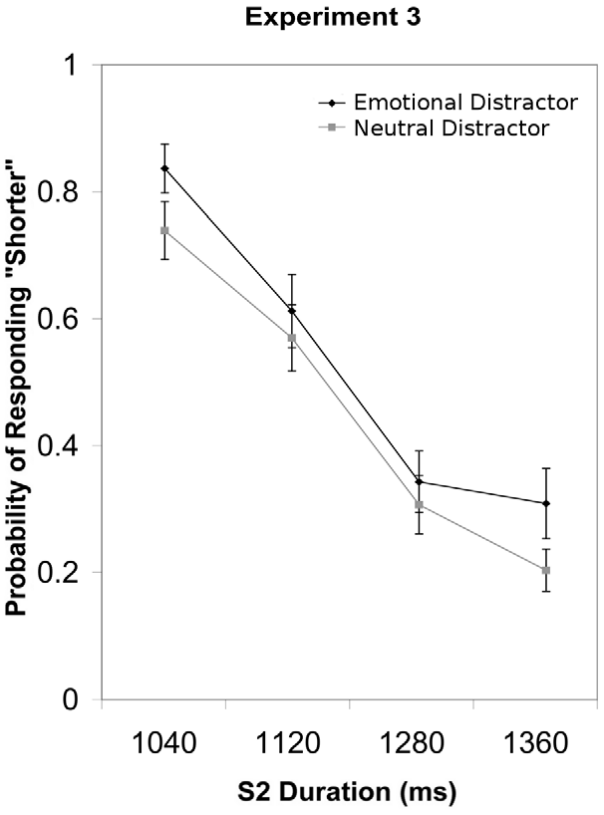
The group mean probability of responding “shorter” and standard error of the mean are plotted for each S2 duration in Experiment 3.

Analysis of the probability of responding “shorter” revealed significant main effects of S2 Duration (*F*(3, 36) = 54.86, *p*<.001, η^2^_partial_ = .821) and Emotion (*F*(1, 12) = 5.28, *p*<.05, η^2^_partial_ = .305). Participants were more likely to respond “shorter” with decreasing S2 duration and when S2 was preceded by a positive as compared to a neutral picture (Fig. 4). S2 Duration and Emotion failed to interact (*p*>.1).

A direct comparison between positive and negative conditions was conducted by subjecting the data from Experiments 1 and 3 to a 3-way Experiment (1 vs. 3)×Emotion (emotional vs. neutral)×S2 Duration (4 levels) mixed design ANOVA with probability of responding “shorter” as the dependent variable. There was a significant S2 Duration effect (*F*(3, 75) = 126.32, *p*<.001, η^2^_partial_ = .835) and a significant Emotion effect (*F*(1, 25) = 14.53, *p*<.01, η^2^_partial_ = .368). Importantly, neither the Experiment main effect nor an interaction with Experiment reached significance (*p*s>.1).

Finally, we explored the contribution of picture valence and arousal to the observed effects in Experiment 1 and 3. IAPS valence scores were converted to emotion intensity scores by subtracting 5 (the rating scale midpoint) from each value and taking the absolute value of the result. A correlation analysis for these and the IAPS arousal scores revealed that both measures were highly correlated (r = .94, p<.0001). We subsequently tested two ANOVA models to determine whether and in what way each measure predicted the probability of responding “shorter” in Experiments 1 and 3. The first model included Emotional Intensity and Experiment as factors and returned a significant effect of Emotional Intensity (F(1,124) = 5.6, p<.05) with all other effects being non-significant (p>.1). The second model included Arousal and Experiment as factors and returned a significant effect of Arousal (F(1,124) = 5.2, p<.05) with all other effects being non-significant (p>.1).

### Discussion

Participants perceived the duration of a stimulus to be shorter when it was preceded by a positive as compared to a neutral picture. Given that this effect was comparable to that observed for negative pictures, one may conclude that positive and negative stimuli similarly affect S2 duration estimation. S2 is perceived to be shorter when immediately preceded by emotional as compared to neutral stimuli. A discussion of non-specific emotion mechanisms by which this effect may occur is presented in the General Discussion.

Below we report a fourth experiment aimed at addressing the question of whether emotion induced changes in attention to time are limited to within modality processing or persist when emotion and timing information come from different modalities. This question is important as attention is a limited resource [35] and emotion effects may spread more readily within than across modalities. Research investigating cross-modal integration found attention to be guided by cross-modal signals [47]–[48]. Cross-modal signals, even if task-irrelevant and uninformative, appear to facilitate the processing of spatially overlapping targets. Existing research further implies that cross-modal influences on attention are stronger for emotional as compared to neutral stimuli [49]–[50]. For example, Brosch and colleagues presented angry and neutral vocalizations dichotically to the left and right ears of participants who were engaged in a visual target detection task. Target detection was enhanced when angry vocalizations were played at the same side as a visual target relative to when they were played at the opposite side. While revealing with respect to cross-modal influences of emotion on attention, this evidence is limited to spatial processing. Moreover, it is unclear what, if any, cross-modal influence would be observed on temporal processing. To address this question, we conducted a fourth experiment in which participants timed the duration of auditory stimuli before and after viewing neutral and negative pictures.

## Experiment 4

### Method

#### Participants

Eighteen female college students participated in the present study in fulfillment of an introductory psychology course requirement. Their mean age was 19.9 years (SD = 0.77). They were all right-handed [39] and reported normal or corrected to normal vision.

#### Procedures

Experiment 4 was equivalent to Experiment 1, with the exception that the timing stimulus (i.e., S1/S2) was a 500 Hz pure tone rather than a circle.

### Results

**Figure 5:**
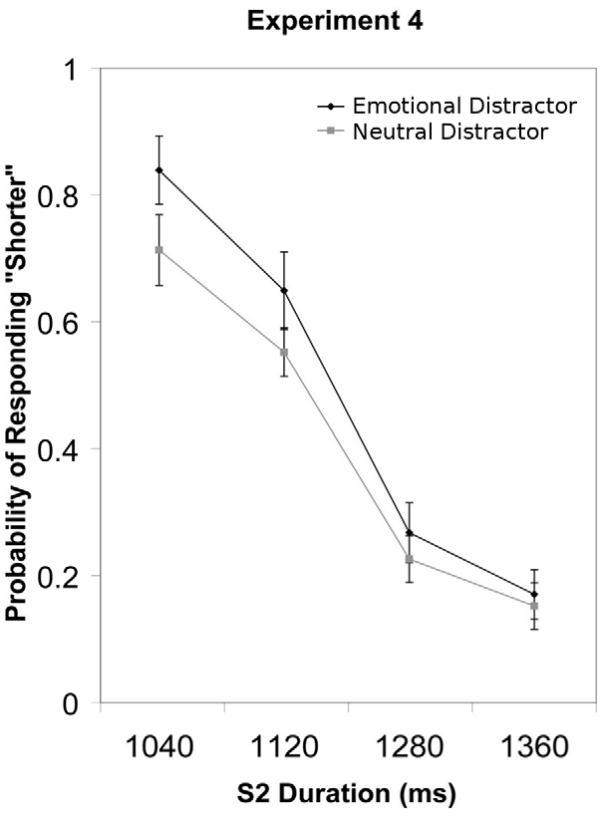
The group mean probability of responding “shorter” and standard error of the mean are plotted for each S2 duration in Experiment 4.

Analysis of the probability of responding “shorter” revealed significant main effects of S2 Duration (*F*(3, 51) = 71.01, *p*<.001, η^2^_partial_ = .807) and of Emotion (*F*(1, 17) = 6.75, *p*<.05, η^2^_partial_ = .284). Participants were more likely to respond “shorter” for shorter S2 Durations and when S2 was preceded by a negative as compared to a neutral picture (Fig. 5). The interaction effect was non-significant (*p>.1*).

### Discussion

In line with Experiments 1–3, participants underestimated the duration of S2 when it was preceded by an emotional as compared to a neutral picture. Given that Experiment 4 used an auditory timing stimulus and manipulated participant emotions through visual stimuli, these results suggest that the effects of emotion on time perception are not limited to within-modality processes, but extend to across modality processes.

Together the current results are opposite to those typically reported in the literature. Emotions here led to temporal underestimation rather than overestimations thus making an attention account a viable competitor of the existing pacemaker account. Nevertheless, one may object that the divergence in results stems from a divergence in methodology, which employed an S1–S2 paradigm in the present case as opposed to the bisection or temporal reproduction paradigms in past research. Therefore, emotion effects on responses may not reflect a shortening of S2 but a lengthening of S1, which was being encoded and maintained in working memory during the emotional challenge. To address this possibility we conducted a final experiment in which each trial comprised the presentation of an emotional or a neutral picture followed by a sample duration, which participants were required to reproduce. If emotions increase the sense of passed and passing time, participants should produce longer durations when primed with an emotional as compared to a neutral image. If, however, emotions distract from the monitoring of time when dissociated from the timing signal, then participants should produce shorter durations when primed with an emotional as compared to a neutral image.

## Experiment 5

### Method

#### Participants

Eighteen female college students participated in the present study in fulfillment of an introductory psychology course requirement. The data of four participants was discarded because they were unable to accurately discriminate the durations presented for reproduction. The mean age of the remaining participants was 22.4 years (SD = 1.86). They were all right-handed [39] and reported normal or corrected to normal vision.

#### Procedures

A trial started with the presentation of a fixation cross, which remained on screen for 500 ms. The cross was followed by a picture presentation for 800 ms. After a short empty interval of 500 ms, a circle appeared on the screen for 1100, 1700 or 2300 ms. After a longer empty interval of 5500 ms, a 500 ms cross prompted participants to attend to the onset of another circle. Participants were asked to push a button when they thought the duration of the second circle matched that of the first circle. Inter-trial intervals were equally distributed among 1800, 2100, and 2400 ms.

The visual stimuli used here were comparable to those of Experiment 1. On half the trials, a timing stimulus was preceded by an emotionally negative picture, whereas on the remaining trials it was preceded by a neutral picture. There were a total of 30 emotional and 30 neutral trials with one third each for the 1100, 1700 and 2300 ms duration conditions. Trials were presented in random order.

### Results

**Figure 6:**
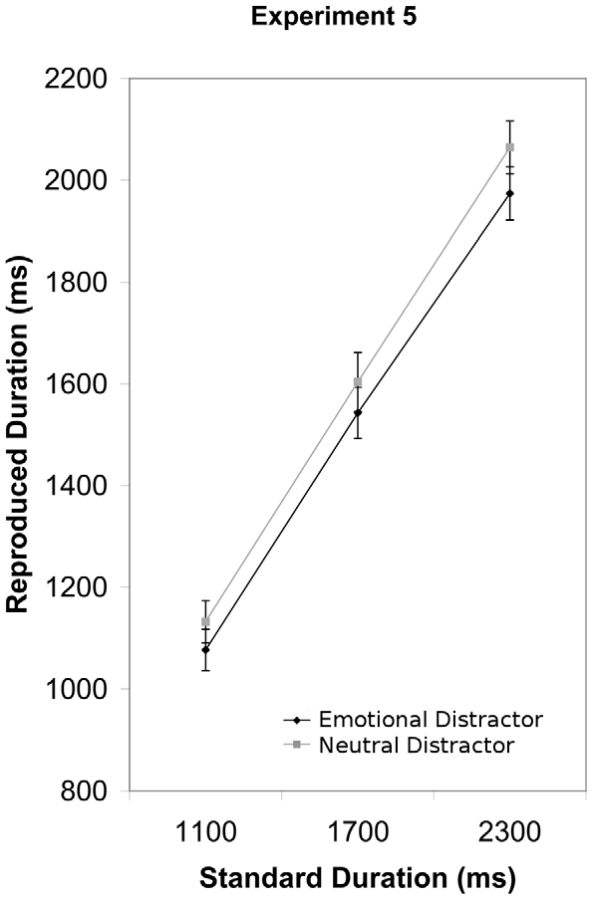
The group mean response time and standard error of the mean are plotted for each target duration in Experiment 5.

We computed mean reaction times and standard deviations for each participant across all experimental conditions and removed trials from the analysis in which participants deviated more than two standard deviations from their respective mean. Using the remaining trials, we then computed means for each participant and condition and subjected those to an ANOVA with Emotion (negative, neutral) and Duration (1100, 1700, 2300) as repeated measures factors. This analysis revealed an Emotion main effect (*F*(1, 13) = 11.23, *p*<.01, η^2^_partial_ = 0.46) indicating that participants reproduced shorter durations when the timing signal was preceded by an emotional as compared to a neutral image. The Duration effect was also significant (*F*(2, 26) = 226.32, *p*<.0001, η^2^_partial_ = 0.94) indicating that participants temporal reproductions were shortest for the 1100 ms condition, intermediate for the 1700 ms condition and longest for the 2300 ms condition (Figure 6). The interaction of Emotion and Duration was non-significant (p>.1).

### Discussion

The subjective shortening of duration when the timing stimulus was preceded by an emotional stimulus in the reproduction paradigm of Experiment 5 confirms that the results of Experiments 1–4 were not merely an artifact of the S1/S2 discrimination paradigm. Moreover, Experiment 5 eliminates the possibility that they were due to emotional events lengthening duration representations that are concurrently maintained in working memory. Rather, emotional events seem to affect temporal processing prospectively. Compared to neutral events, emotional events shorten the perception of subsequently presented timing signals.

## General Discussion

The present study set out to investigate the influence of emotions on time perception. Of particular interest was whether pacemaker rate or attention represents a more viable mechanism for emotional effects on timing. Across five experiments, we found that participants perceived a timing stimulus as shorter when it was preceded by an emotional as compared to a neutral distracter. Moreover, this effect was observed for within modality and across modality manipulations, for positively and negatively valenced emotional distracters, for 500 ms and 2000 ms intervals between the distracter and the timing stimulus, and was independent of the type of timing response (i.e., temporal discrimination or reproduction) participants made.

Together these results challenge the idea that emotions influence time perception merely by influencing pacemaker rate. If that were the case, then the present study should have replicated prior work indicating that participants overestimate the duration of emotional as compared to neutral events [15]–[19]. That is to say, the emotional distracters in the present study should have increased pacemaker rate and allowed more pulses to be accumulated. Compared to the condition with neutral distracters, this should have reduced the proportion of “shorter” responses to the S2. As we found the opposite pattern of results, a mechanism other than pacemaker rate likely accounts for our effects. We propose attention to be this mechanism and in the following paragraphs suggest some possible ways by which emotions might alter attention to affect changes in time perception.

First, it is possible that participants directed greater attention to the encoding of emotional as compared to neutral pictures and thus tended to miss the onset of the timing stimulus. In support of this, we found the emotion effect to be statistically independent of timing stimulus duration. However, the durations used here may not have been different enough and/or the statistical power too small to detect duration related changes in the emotion effect. In support of this, we found that emotion induced underestimation was unaffected by an increase in the interval between the emotion stimulus and the timing stimulus in Experiment 2. This increase should have reduced an underestimation effect due to a delayed latency to begin timing because participants had more time to recover their attention. Moreover, in Experiment 5 participants reproduced shorter durations in the emotion condition despite the fact that they were prompted about the impending reproduction of the timing stimulus. Finally, the condition means in the latter experiment suggest that the temporal shortening effect increases from the short (56 ms), to the intermediate (61 ms) to the long duration condition (91 ms). Thus, there are likely other attentional mechanisms contributing to the present results besides a mere onset effect.

One such mechanism could involve the effect of non-specific bodily arousal on attention. Previous research has indicated a relationship between bodily arousal and cognitive performance, which is commonly known as the Yerkes-Dodson law [51]. According to this law, cognitive performance increases with increasing arousal up to an optimal level. Once this arousal level is passed, cognitive performance declines. Although the Yerkes-Dodson law has been criticized [52], past findings were generally supportive making it a candidate explanation for emotion effects on timing. Accordingly, the mix of moderately and highly arousing images used here may have led to a general cognitive impairment that included attention and thus impacted participants' ability to monitor time. This assertion is supported by the fact that temporal judgments were related to arousal. However, as they were equally strongly related to emotion intensity, bodily arousal may not or not solely account for the observed effects.

A final mechanism we would like to propose involves attention capture by appraisal processes and/or emotion regulation attempts [53]–[54]. Preoccupation with such processes could deter participants from focusing on the timing task. Neuroimaging research revealed an involvement of lateral and medial prefrontal cortex in both emotion appraisal [55] and emotion regulation [56]. Given that similar substrates have been implicated in the perception of time [57]–[58], emotion regulation might compete with time perception for these neural resources.

Although our data support an attention account, one may object that a pacemaker rate account cannot be fully ruled out. This is because participants may have used the emotional/neutral picture as a reference for judging or reproducing the target duration. As emotional stimuli were found to be overestimated in previous studies, a longer timing reference may have led to an underestimation of the target. However, the following consideration makes this possibility implausible. Changes in arousal after an emotional challenge develop in time and increases in arousal markers such as heart rate and skin conductance have been noted to peak between 3 to 6 seconds following stimulus onset [59]–[60]. Given the interval between picture onset and the imperative timing signal varied from 1.3 to 2.8 seconds across the present experiments, an arousal effect should have been smaller during picture processing than during S2 processing and thus increased the probability of ‘longer’ responses. However, we observed the opposite result indicating that pacemaker rate is ill-suited to explain the relationship between emotion and time. Instead, attention capture by the emotional distracter appears more appropriate.

Although the pacemaker account has been very popular as an explanation of the relationship between emotion and time, the present work is not the first to suggest a relationship between emotion, attention, and time. For example, Meck and MacDonald [24] observed that lesions to the amygdala, a structure known to mediate the attentional bias towards emotional events, caused a deficit in the temporal estimation of threat stimuli. This deficit was comparable to that caused by lesions to the prefrontal cortex, a structure implicated in attentional control [24]. Furthermore, a study by Gil and colleagues [23] found time estimates to be shorter for disgusting food pictures as compared to neutral pictures. Unlike threat-relevant stimuli, disgusting stimuli are disease salient and only harmful if we approach them. Thus, rather than eliciting a fight-or-flight response, they simply warn us to stay away [61]. Therefore, Gil and colleagues [23] argued that the perception of disgusting stimuli causes attentional diversion and temporal pulses to be lost.

Although prior work cited above raises the possibility that attention capture or aversion modulate the relationship between emotion and time, it does not exclude a pacemaker account. Apart from modulating attention, lesions to the amygdala are known to impair arousal responses to emotional stimuli [62]–[63], which offers an alternative explanation for the timing impairments seen by Meck and MacDonald [24]. Moreover, in the study by Gil and colleagues [23], disgust was likely associated with a reduction in arousal. Heart rate, a key marker for arousal, has been shown to slow down during disgusting experiences [64] and thus the temporal underestimation of disgusting images could be due to pacemaker rate decreasing and fewer temporal pulses being accumulated. Given that these interpretive difficulties do not apply to the present work, it can be considered a meaningful extension of the literature on emotion, attention, and time.

### Conclusion

The present set of experiments demonstrates that individuals underestimate the duration of a neutral stimulus if that stimulus is presented in an emotional as compared to neutral context. This is true regardless of whether the emotion is positive or negative suggesting that both positive and negative experiences can affect timing in similar ways. The mechanism by which this occurs is unlikely to involve the pacemaker, but instead may arise from a modulation of attention. If true, this would lend a parsimonious explanation of why emotions can both slow down and speed up everyday experiences. A slowing down of perceived time may occur when individuals wish for an experience to end quickly and hence direct resources towards monitoring the passing of time. In contrast, a speeding up of time occurs when the experience captures attention leaving the passing of time unnoticed and leading us to believe that “time flies”.
